# Scraps

**Published:** 1900-03

**Authors:** 


### SCRAPS

PICKED UP

BY L. M. G.

---

**From the Seat of War.**—An old-fashioned doctor of this neighbourhood when he heard that Lady Smith had been relieved said that he hoped the *primæ viæ* would be kept open

**Clinical Records** (30).—Mother: "What did you do with that medicine the doctor left for you?" Small Boy: "I heard there was a poor boy ill in the back street, an' I took it round and left it for him."

**Zulu Medical Lore.**—The Under-Secretary for Native Affairs in Natal, in a lecture on "Zulu Social and Domestic Life," gave some interesting particulars concerning certain prescriptions of the Zulu medicine men:—If you have the mumps, go to an ant-bear's hole, look into it, and call out "zagiga! zagiga!" (let me alone), and if you return home without looking back, the mumps will leave you. If you are deaf, get a monkey's ear, burn it to ashes, mix the ashes with hippopotamus fat and beeswax, drop some of the mixture into your ears, and your hearing will soon be as good as the monkey's.

**The New Anatomy.**—*Practical Medicine* and *The Student* have collected from the newspapers some instances of the newer (literary) anatomy, from which I quote the following:—The murderers have discovered some astonishing vulnerable parts of the human anatomy of late; a Georgia colonel was shot in the ticket-office, the other day a man was fatally shot through his door, and not long ago another received a fatal wound in his window. A young man meeting his loved one after some interval kissed her passionately upon her reappearance. A mother irritated by the long delay of her son, who had been sent on a message, whipped him upon his return. A young lady seeking an interview with the editor sat down upon her being asked. A poor woman was shot in the oil regions some time ago; and another more recently was accidentally shot in the waterworks; while an unfortunate man was shot in the suburbs. A woman who sat down upon the spur of the moment was none the worse for it.

**The Whole World Kin.**—Many English doctors may profit by the lessons contained in a recent editorial in *The Cincinnati Lancet-Clinic*, from which these extracts are taken:—"The time was, and is not so very far back, when the ordinary general practitioner of medicine thought he was doing entire justice to himself, his profession and his clients if he took a single medical journal. Conditions and times have greatly changed within the last twenty years . . . so that the men who are ambitious to be at the front find themselves obliged to purchase new and improved instruments of precision, new books, and to take more than one or two medical journals. . . . The busiest men in the medical profession always attend the National, State and local medical societies. They are the ones who read papers, take part in the discussions, and then attend all the social functions. . . . The men who can't get off to attend such meetings are worried and perplexed for fear some rival will profit by their absence, instead of swapping time and business with such rivals, through which both would prosper and be better thought of. The world is wide, and there are other pebbles on the beach."

**Medical Philology** (XXXIII.).—In the *Promptorium* "Dyhtyñ" is given as the equivalent of "*Paro, preparo.*" This was one of the many forms of spelling of the later and now almost obsolete "dight." Its various meanings are well illustrated in the *New English Dictionary*; that in the *Promptorium* was one that was frequent.

Mr. Way's note is: "In the Household Book of Sir John Howard, A.D. 1467, among expenses incurred for one of his retinue, is entered this item, 'My Lady paid a surgeone for dytenge of hym, whan he was hurte, 12*d.*' Palsgrave gives the verb in its more usual sense, 'to dyght, or dresse a thynge, *habiller*. A foule woman rychly dyght, semeth fayre by candell lyght.' Ang. Sax. dihtan, *disponere*." The "dytenge" which the member of Sir John Howard's household received after his injury was probably a very small piece of minor surgery, and may have been no more than the dressing of a slight wound. In the *New English Dictionary*, which gives several instances of the use of the word to signify some surgical attention, there is this quotation, derived from that which is described as: "*Mann. & Househ. Exp.*, 1467." "My wyffe payd to a schorgon, fore dytenge of heme wane he was horte, xij.d." I am not able to give in full the authority from which this quotation is taken, but I presume it refers to the same incident which Mr. Way mentions in his *Promptorium Parvulorum* note. The spelling "schorgon" is extraordinary, and was probably never an accepted form.

**Tuberculosis.**—Some time ago the suggestion was made in all seriousness that the Underground Railway was a Sanatorium for chest complaints. *Truth* offered a prize for rhymed comments on this suggestion. From a collection of verses sent in on the subject I take this, which appeared over the pseudonym of "Almaviva":

Ho! all ye invalids who have a weakness of the chest,
Whose lungs are full of microbes, and whose coughing knows no rest,
We've joyful news for you indeed, a remedy is found,
'Tis close at hand, and very cheap, in fact, the "Underground."
Just travel round the "Circle," and inhale its beastly fumes,
And all the microbes take affright, and health her sway resumes.
Mayhap you live too far away, then this will meet your need:
Look out a cellar, damp and dark, and very close indeed,
Then fix a grate or brazier there, in which you then must poke
A small amount of sulphur and a good supply of coke;
A kettle you must place on top, to make the needful steam,
Then light your fire, and soon that place the Underground will seem.
If every day for several months those fumes you just inhale,
You'll be as sound as any bell, and red instead of pale;
Though some may think, when they've so much discomfort to endure,
That, after all, their old complaint is better than the cure.

*The Practitioner* for this month says that Dr. Walther of Nordrach owes his success in the treatment of consumption largely to the fact that he is what Jeames would call a "harbitrary gent." The following lines are by a well-known actor, who has been under his care, but whose name we are not at liberty to disclose. They give a vivid picture of the Nordrach treatment from the patient's point of view:—

"THE AUTOCRATIC DOCTOR."

[*With acknowledgments to Rudyard Kipling.*]

I.

When you've swallowed Scott's Emulsion by the gallon or the jug,
  When you've finished Iodinin' of your back,
Will you kindly drop your sputum in my little china mug
  And send it to a party at Nordrach?
He's an autocratic doctor with a rough and ready tongue,
  But Tubercular Bacilli can't abide him,
And the patient finds him busy wiping something off his lung
  By cramming lots of little things inside him.

Raw meat, cooked meat, meat of a hundred kinds,
  Fifty chronics at table, striving to eat their lunch,
Each of 'em doing his level best to swallow the skins and rinds.
  Pass your plate for credit's sake and munch, munch, munch!

II.

There are some who "pouch" in secret, asking no permission to,
  For they know they wouldn't get it if they did,
Scraps of cheese, and bits of lobster, lumps of meat they couldn't chew,
  And a rather more than "gamey" piece of kid.
And havin' been so casual, they feel sorry when they're gone
  (For the Autocratic Doctor's sure to "out" 'em).
When their lungs are going dicky with the winter coming on
  They'll miss the bloke who understood about 'em.

Cooked food, raw food, plenty of milk and rest,
  Quarter o' pound o' butter—*Schwarzbrod* by the hunch,
Each of 'em tryin' to raise his weight and widen his girth and chest.
  Pass your plate for your credit's sake and munch, munch, munch!

